# A large-scale dataset of *in vivo* pharmacology assay results

**DOI:** 10.1038/sdata.2018.230

**Published:** 2018-10-23

**Authors:** Fiona M. I. Hunter, Francis L. Atkinson, A. Patrícia Bento, Nicolas Bosc, Anna Gaulton, Anne Hersey, Andrew R. Leach

**Affiliations:** 1 European Bioinformatics Institute (EMBL-EBI), Wellcome Genome Campus, Hinxton, Cambridge CB10 1SD, United Kingdom

**Keywords:** Experimental models of disease, Translational research, Drug development, Drug discovery and development, Toxicology

## Abstract

ChEMBL is a large-scale, open-access drug discovery resource containing bioactivity information primarily extracted from scientific literature. A substantial dataset of more than 135,000 *in vivo* assays has been collated as a key resource of animal models for translational medicine within drug discovery. To improve the utility of the *in vivo* data, an extensive data curation task has been undertaken that allows the assays to be grouped by animal disease model or phenotypic endpoint. The dataset contains previously unavailable information about compounds or drugs tested in animal models and, in conjunction with assay data on protein targets or cell- or tissue- based systems, allows the investigation of the effects of compounds at differing levels of biological complexity. Equally, it enables researchers to identify compounds that have been investigated for a group of disease-, pharmacology- or toxicity-relevant assays.

## Background & Summary

**Figure 1:**
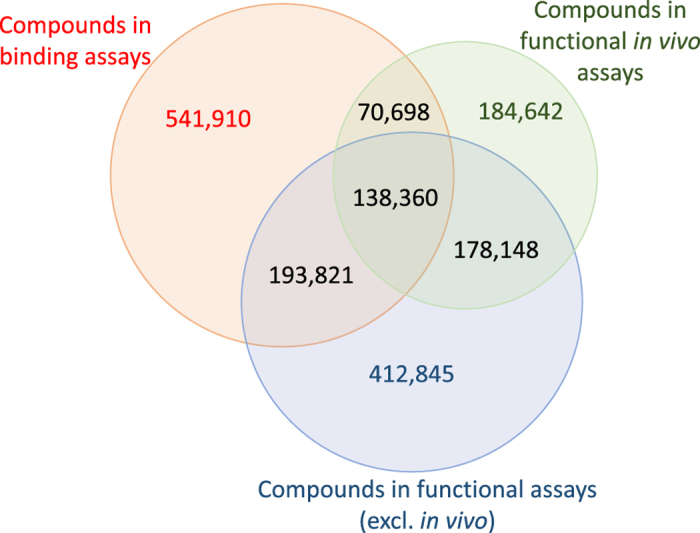
Venn diagram of the number of distinct compounds across ChEMBL (version 24), classified by the biological complexity of the assay system. The assays have been grouped using the assay_type: B (binding) which represents interaction of compounds with molecular targets; F (functional) *in vivo* (defined by BAO_0000218 - organism-based format) and non *in vivo* functional assays (ie those in cell-, tissue- or organ-based systems), and the number of distinct compounds in each assay group were counted regardless of their activity (or inactivity), biological target or units of measurement.

ChEMBL (https://www.ebi.ac.uk/chembl) is a large-scale, open-access drug discovery resource containing information about bioactive molecules, their interaction with targets (*e.g.* molecular, cell- or tissue-based) and their biological effects^1–4^. It aspires to the FAIR data management principles (Findable, Accessible, Interoperable, and Reusable)^5^. ChEMBL is uniquely positioned to study the translation between assays that investigate differing scales of complexity, from the molecular scale that considers binding of compounds onto individual protein targets through to disease-relevant outcomes carried out on whole organisms. This approach is analogous to the Adverse Outcome Pathway framework^6,7^ (https://aopwiki.org/) that attempts to link between a molecular initiating event and a higher level response such as an adverse effect on a cell, organ or organism. For example, ChEMBL contains around 280,000 *binding assays* that investigate the bioactivity of a compound or an approved drug on a protein target (for ~945,000 distinct compound structures). Equally, ChEMBL also contains around 550,000 *functional assays* that investigate the biological effect of an individual compound within the increasing complexity of a cell-, tissue-, or organ-based system (for ~570,000 distinct compound structures), or within a whole animal disease model (for ~920,000 distinct compound structures). For example, functional assays may examine the percentage of cell death in a cell line, or the inhibition or change of a response within a whole animal disease model. If all biological targets are considered, ChEMBL contains around 138,000 distinct compound structures that have been tested in binding assays as well as cell-, tissue-, or organ-based systems and whole animal disease models regardless of their activity (or inactivity) or units of measurement (Fig. 1). In addition, ChEMBL also contains around 200,000 assays (for ~210,000 distinct compound structures) that investigate the effect of the organism on a compound through Absorption, Distribution, Metabolism and Elimination (ADME) studies which includes *in vivo* pharmacokinetic data. Given the range of pharmacological data at varying scales of biological complexity, ChEMBL provides a rich, high-quality resource for addressing a wide range of drug discovery-related questions.

One key aspect of pre-clinical drug discovery is the testing of potential therapeutic compounds in animal safety models to understand disease or phenotypic outcomes and assess the potential for toxicological or adverse effects. An animal model can provide a realistic and predictive measure of the effect of a compound in a biologically complex system such as a clinical outcome in human patients. Despite significant ongoing work to reduce the use of laboratory animals^8^ and develop integrated in silico tools to predict human liver and heart toxicity (*e.g.* Kuepfer *et al.*^9^ or Passini *et al.*^10^), regulatory agencies typically require proof of compound safety in animals before progressing a potential drug into clinical studies in human (*e.g.* FDA guidance for Phase I studies^11^). Therefore, there is much value for data users to be able to access well-organised and clearly annotated *in vivo* assay information on relevant animal studies.

Recent work has applied natural language processing to mine the ChEMBL *in vivo* assay descriptions for relevant information such as experimental treatment and phenotypic outcomes^12^. They demonstrated that annotated *in vivo* assay information can provide insights into inter-relationships between experimental models, drugs and disease phenotypes^12^.

The *in vivo* assay data within ChEMBL is likely to be under-utilised due to:

- its unstructured format that comprises a textual description of the assay along with measured endpoints and units of measurement that are frequently non-standard;
- its relatively complex nature in comparison to biochemical screening data that examines the effect of one compound on one protein target. For example, an *in vivo* assay might describe a chemically-induced phenotype such as carrageenan-induced oedema in the paw of a rat and the effect that a test compound has on the oedema, or the assay may describe the effect of a test compound in a rat to block a seizure that had been induced by an electric shock; and
- the lack of a standard annotation to organise similar categories of *in vivo* assays.

**Figure 2:**
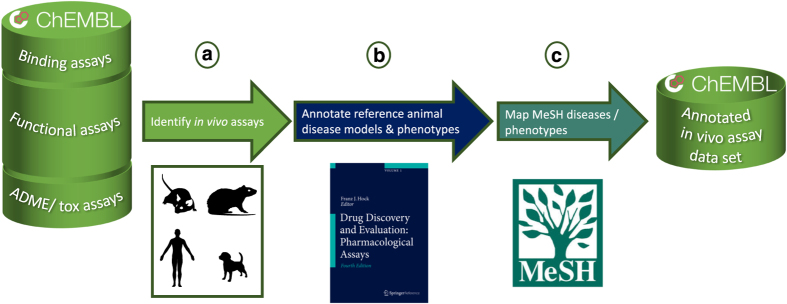
The workflow to identify and annotate the *in vivo* assay dataset. (**a**) The *in vivo* assays within the ChEMBL database are identified. (**b**) The assays are annotated by reference animal models described by the Hock publications and/or by a disease or phenotypic endpoint with pharmacological or toxicological relevance. (**c**) The reference animal models or disease/phenotypic endpoints are mapped to MeSH terms.

A dataset of *in vivo* assays has been collated from ChEMBL and annotated by reference animal disease models or phenotypic endpoints that have pharmacological or toxicological relevance (Fig. 2a,b). A second layer of annotation has mapped Medical Subject Heading (MeSH) disease terms to improve the interoperability of the *in vivo* assay dataset (Fig. 2c). The resulting dataset will allow increased usage of the *in vivo* assays and their associated disease, phenotype and toxicity information. For example, using the new annotation, a subset of the *in vivo* assay dataset that considers Parkinson’s disease can now be collectively examined for similar patterns. Likewise, *in vivo* assays that investigate, for example, animal models of pain or hepatotoxicity can be collectively examined.

In this way, the work provides a significant step forwards in the organisation, annotation and accessibility of the *in vivo* assay dataset, resulting in a defined dataset of *in vivo* assays and their associated information such as the disease area or phenotype for which the assay has been investigated. The *in vivo* assay dataset and its associated information has been implemented in ChEMBL so that it can be utilised in a structured way and can be linked to other relevant data in a straightforward manner. The dataset has the potential to be used to identify new tool compounds, new indications for repurposed drugs, or to uncover as yet unidentified off-target effects or other toxicological effects in the pursuit of safer medicines.

## Methods

### *In vivo* assay identification

The set of *in vivo* assays has been collated from ChEMBL (version 24) using the BAO Ontology^13,14^ that typically categorises *in vitro* screening assays but can also be used to distinguish assays that are performed *in vivo* (‘organism-based format’) from *in vitro*, *ex vivo* assays *etc*. Then, to identify *in vivo* assays that consider animal models (*e.g.* for Rat, Mouse or larger mammals) rather than insects, bacteria, viruses *etc*, a second step was required to separate relevant *in vivo* assays from other *in vivo* assays using ‘mammals’ as the annotated organism class of the assay or target. This process is considered to be a relatively clean method to separate the *in vivo* assays that investigate animal models from other functional *in vitro* or *ex vivo* assays. Note that some *in vivo* assays may xenograft a human cell line into a mouse animal model, in which case the assay organism would be described as Mouse while the target organism would be described as Human. An alternative approach to use the ‘F’ assay type to extract all functional assays, followed by the ‘*in vivo*’ assay test type was considered but this gave less comprehensive results because the ‘assay test type’ is a less well populated database field in ChEMBL. In addition, assays that investigate ADME processes have been excluded since these relate to the measurement of pharmacokinetic properties or *in vitro* drug metabolism studies rather than disease-, phenotype- or toxicity-relevant animal models. Equally any assay description that contains a reference to an *in vitro* or *ex vivo* assay has been excluded.

Each of the identified *in vivo* assays has a compact, free-text assay description that was created by the data extractor when the information was added to ChEMBL^1–4^. Note that the extraction of assay descriptions into ChEMBL was not carried out as part of the work described in this article. Despite the absence of a formal controlled vocabulary, there are many common text patterns contained within each assay description. The free text assay descriptions that are available in ChEMBL have never previously been curated or organised into a defined dataset. The *in vivo* assay descriptions vary in vocabulary, syntax and length but often contain phrases that identify an animal reference model, or a specified phenotypic endpoint, or both, although in some cases the assay description is too sparse to identify a unique animal reference model or a phenotypic or toxicological endpoint, especially in data described by early versions of ChEMBL (examples are given in Table 1).

### *In vivo* assay annotation

There is no existing ontology or controlled vocabulary that attempts to categorise disease-, phenotype- or toxicity-relevant animal models. For example, ontologies exist to describe phenotypic outcomes observed in animal models (*e.g.*^15,16^), but not the animal models themselves. Therefore, to improve the organisation and accessibility of the identified *in vivo* assay dataset, an annotation task has been carried out based on:

- published information available in a set of reference books that comprehensively describe pharmacological and safety assays (Hock publications – see below), and
- observation of common phrases within each assay description that identify a disease or phenotypic endpoint with pharmacological or toxicological relevance.

The assay annotation has been structured such that each identified *in vivo* assay in the dataset can be assigned an assay classification (at level 3) if possible, as well as subsequent annotation at two higher levels (level 2 and level 1). Due to the absence of an existing ontology that describes range of available animal and safety models, this annotation approach is regarded as a significant and consistent forward step to improve the utility of the data.

The comprehensive reference works are (i) ‘Drug Discovery and Evaluation: Pharmacological Assays’^17,18^ (edited most recently by Hock in 2016) which describes many functional assays in substantial detail and (ii) ‘Drug Discovery and Evaluation: Safety and Pharmacokinetic Assays’^19^ (edited by Vogel). Hock^17,18^ or Vogel^19^ describe around 1100 pharmacological and safety pharmacological models that may be classed as functional *in vitro*, *ex vivo* or *in vivo* (thereafter these publications are collectively referred to as ‘Hock publications’). For each reference model, the Hock publications provide an assay name, purpose and rationale, procedure, evaluation, critical assessment of the method, modifications of the method, references and further reading. In addition, similar assays are organised by chapter. For example, the Hock^17,18^ chapter on “Cardiovascular Analysis *in vivo*” contains reference animal models that investigate blood pressure by different methods, angiotensin II antagonism for hypertension treatment or the Bezold–Jarisch reflex that causes excessively shallow breathing or an abnormally low resting heart rate, while Vogel^19^ describes reference animal models of cardiovascular safety pharmacology such as blood pressure or cardiac output.

The first stage of the annotation approach has been to find a text pattern that uniquely identifies a reference animal model and to match this pattern against the text contained within the ‘description’ field of the *in vivo* assay dataset (Table 1). For example, the regular expression ‘[Tt]ail\W?[Ff]lick’ identifies the ‘Tail Flick’ reference animal model described by Hock, and allows the annotation of all *in vivo* assays that have a relevant assay description *e.g.* “Analgesic activity in tail flick test, oral administration” (CHEMBL732290), or “Compound was administered subcutaneously and was evaluated for opioid antagonist activity (versus morphine) by tail-flick (TF) antagonism test” (CHEMBL723844) (Table 1). The text patterns have been manually assigned, and a positive (and negative) check of the resulting assay hits was carried out. A text pattern match to uniquely identify an individual reference animal model has been created for around half of the *in vivo* animal models described by the Hock reference works. The remaining animal models described in the Hock reference works either relate to an *in vitro* or *ex vivo* experiment, or an *in vivo* animal model that cannot be uniquely identified by phrases that may be contained within the assay description. For example, ‘MRI Studies of Cardiac Function’ or ‘Chronic Stress Model of Depression’ are animal models that require multiple experimental observations, some of which overlap with experimental observations for other animal models and therefore a text pattern match within an assay description from the *in vivo* dataset does not uniquely identify one specific animal model. For this reason, of the 514 *in vivo* animal models described in the Hock reference works, around half (260 animal models) could not be mapped to any assay description within the *in vivo* assay dataset.

If applicable for each reference animal model, a compound that induces a phenotype in the reference animal model is recorded (*e.g.* carrageenan or formaldehyde are used to induce paw oedema in rat). Equally, any standard ‘positive control’ compound that causes a known result for a reference animal model is noted (*e.g.* morphine, codeine or meperidine are positive control compounds for the ‘Tail Flick’ reference animal model).

The second stage of the annotation approach is as follows. For some *in vivo* assays, a disease or phenotypic endpoint with pharmacological or toxicological relevance can be identified from the assay description. For example, the assay description given as (Table 1):

“Antioxidant activity against CCl4-induced oxidative hepatic injury Wistar albino rat model assessed as effect on liver cytosolic catalase activity per mg protein at 100 mg/kg, ip for 7 consecutive days prior to CCL4 challenge measured 24 hrs post CCl4 challenge (Rvb = 218.25 +/− 11.43 U/mg protein)” can be annotated by a general toxicological endpoint (‘General Models of Drug Induced Liver Injury’) as well as a specific reference animal model (‘Carbon tetrachloride CCl4 Induced Liver Fibrosis in Rats’).

**Figure 3:**
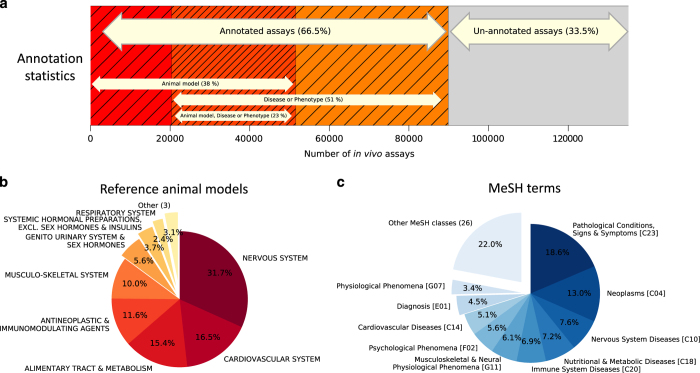
The *in vivo* assay dataset and its annotation. (**a**) Of the total number of identified *in vivo* assays, 89 844 assays (66.5%) have been annotated, while 45 347 assays (33.5%) remain unannotated. Of the annotated *in vivo* assays, 51 580 assays (38%) have been annotated by a Hock^17,18^ or Vogel^19^ reference animal model, 69 449 assays (51%) have been annotated by a disease or endpoint with pharmacological or toxicological relevance, and 31 185 assays (23%) have been annotated by both reference animal model and a disease or phenotypic endpoint. Note that some assays are annotated by more than one reference animal model or disease or phenotypic endpoint. (**b**) Breakdown of the annotated animal models for *in vivo* assays by frequency of occurrence and grouped at level 1 assay classification headings. Low frequency groups of assay classification level 1 headings are (<2%; ‘Other’): ‘Blood and Blood Forming Organs’, ‘Sensory Organs’ and ‘Dermatologicals’. (c) Breakdown of the annotated *in vivo* assays by frequency of occurrence of MeSH terms (level 1, e.g. ‘C04’). Low frequency groups of MeSH level 1 terms (<3%; 26 level 1 classes) have been grouped together; this includes, ‘Behavior & Behavior Mechanisms [F01]’, ‘Mental Disorders [F03]’, ‘Endocrine System Diseases [C19]’, ‘Circulatory & Respiratory Physiological Phenomena [G09]’ and ‘Respiratory Tract Diseases [C08]’.

The number of annotated and unannotated *in vivo* assays and a breakdown of their statistics are shown in Fig. 3a. The annotated *in vivo* assays have been grouped by similar animal reference models at the level 1 assay classification (Fig. 3b). This shows that the many of the annotated animal models for *in vivo* assays investigate the nervous system (32%), or the cardiovascular system (17%). These proportions reflect the types of phenotypes that lend themselves to investigation by animal models and are described within the *in vivo* assay dataset. The unannotated *in vivo* assays typically have assay descriptions that are too sparse or non-specific to be able to identify a unique animal model or disease or endpoint with pharmacological or toxicological relevance. Examples of assay descriptions and their annotation (or lack of annotation) are given in Table 1.

Looking forward, there may be opportunities to refine the annotation of the *in vivo* assay dataset as additional assays are identified within future releases of the ChEMBL database, and/or new reference animal models are developed. However, it is likely that some *in vivo* assay descriptions within the identified dataset will remain unannotated unless substantial effort to investigate the underlying published literature source(s) is performed.

### Disease and phenotype mapping (with MeSH)

To improve the interoperability of the *in vivo* assay dataset, a second annotation task has been performed that provides mapping of relevant Medical Subject Heading terms (MeSH, version 2018; https://www.nlm.nih.gov/mesh) to each reference animal model, or disease or phenotypic endpoint with pharmacological or toxicological relevance. Examples are given in Table 2. MeSH is a comprehensive controlled vocabulary of medical terms that can been applied to translational drug discovery because it includes branches for relevant high-level categories like Disease (C) or Mental Disorders (F03) as well as their underlying terms. MeSH have been selected for the second layer of annotation because:

- MeSH terms have good interoperability with commonly used ontologies or controlled vocabularies such as the Disease Ontology (DOID), Human Phenotype Ontology (HPO), Monarch Disease Ontology (MONDO), Unified Medical Language System (ULMS) and EFO (Experimental Factor Ontology), with identifier mapping provided by e.g. EMBL-EBI Ontology Xref Service^20^ (OxO; https://www.ebi.ac.uk/spot/oxo).
- there are existing datasets within ChEMBL that use MeSH to describe e.g. disease indications for approved drugs. Note that >90% of the disease indications in ChEMBL are mapped to both MeSH and EFO terms (although the definitions of these terms may not be exact matches);
- MeSH terms are used to annotate relevant disease terms for clinical studies described by e.g. ClinicalTrials.gov

Therefore, annotation of the *in vivo* assay dataset by MeSH terms allows similar information to be translated across the varied datasets that are used within the drug discovery pipeline.

The MeSH annotation provides a link between a disease or phenotypic outcome and an underlying *in vivo* assay or group of *in vivo* assays. Figure 3c provides a breakdown of high-level categories of annotated MeSH terms for the *in vivo* assay dataset, and shows that many of the annotated *in vivo* assays can be mapped to MeSH terms (at level 2) for ‘C23: Pathological Conditions, Sign and Symptoms’ (18%; includes *e.g.* ‘inflammation’, ‘seizures’, ‘pain’, ‘obesity’), ‘C04: Neoplasms’ (13%; includes *e.g.* ‘neoplasms’, ‘leukemia’, ‘carcinoma’, ‘melanoma’), ‘C10 Nervous System Diseases’ (7%; includes *e.g.* ‘seizures’, ‘memory disorders’, ‘parkinson disease’), ‘C20 Immune System Diseases’ (7%; includes ‘diabetes mellitus’, ‘immune system diseases’, ‘asthma’) or ‘C18: Nutritional and Metabolic Diseases’ (13%; includes *e.g.* ‘lipid metabolism disorders’, ‘diabetes mellitus’, ‘nutrition disorders’). Note that an individual reference animal model can be mapped to more than one MeSH term, and that a MeSH term can be described within more than one MeSH class at level 2. Therefore, the frequency of related categories is not necessarily similar (*e.g.* 12% of animal models investigate antineoplastic and immunomodulating agents in Fig. 3b compared to 13% MeSH terms for Neoplasms in Fig. 3c).

### Code availability

Scripts have been made available (at https://github.com/chembl/chembl_invivo_assay) to carry out:

- the identification of the *in vivo* assays (SQL script, see following subsection; and at github),
- the annotation of the *in vivo* assay dataset by reference animal model, by disease or phenotypic endpoint with pharmacological or toxicological relevance, and by MeSH terms (Python 3 script; at github)

Using these scripts, other researchers can reproduce how the *in vivo* assay dataset has been identified and, in conjunction with the assay classification table that includes manually assigned text patterns (available at github), perform annotation of the *in vivo* assay dataset.

### SQL query used to extract *in vivo* assays from ChEMBL


SELECT DISTINCT a.chembl_id as assay_chemblid, a.description as
            assay_description FROM assays a -- First find ASSAY_organisms that are mammals by
            joining target_dictionary and organism_class: JOIN target_dictionary b ON a.assay_tax_id
            = b.tax_id JOIN organism_class c ON b.tax_id = c.tax_id -- Second find TARGET_organisms
            that are mammals by joining target_dictionary and organism_class: JOIN target_dictionary
            d ON a.tid = d.tid JOIN organism_class e ON d.tax_id = e.tax_id -- Keep assays where the
            BAO Ontology (BAO_0000218) is "organism-based format" WHERE a.BAO_FORMAT = 'BAO_0000218'
            -- Keep assays where either the ASSAY_organism OR the TARGET_organism are mammals. This
            excludes bacteria, insects etc that are also classed as whole organisms: AND (c.l2 =
            'Mammalia' OR e.l2 = 'Mammalia') -- Exclude assay descriptions that relate to in vitro
            or ex vivo assays (assumes all assays have an assay description): AND NOT
            REGEXP_LIKE(lower(a.description), 'in[ -]?vitro|ex[ -]?vivo', 'i') -- Exclude ADMET
            assays since these typically relate to pharmacokinetic parameters like Cmax, Tmax,
            Bioavailability or in vitro drug metabolism studies, and are therefore not disease or
            phenotypic assays: AND a.assay_type != 'A' -- Only include assays from published
            scientific literature. This excludes deposited datasets like TG-GATES that have existing
            annotation. AND a.src_id = 1;


## Data Records

The dataset consists of a collection of around 135,000 *in vivo* assays that relate to disease-, phenotype- or toxicity-relevant animal models and have been typically been performed on target organisms such as Rat (45%) and Mouse (37%) as well as Human (5%), Dog (4%), Guinea Pig (4%), Rabbit (2%) and other mammals (3%). There are ~93,000 distinct compound structures associated with the ~90,000 annotated *in vivo* assays (Fig. 3). The identified *in vivo* assay dataset originates from around 14,600 scientific literature articles that are mainly published by medicinal chemistry journals such as the Journal of Medicinal Chemistry or Bioorganic & Medicinal Chemistry Letters and have had relevant drug discovery information extracted and manually curated as part of the ChEMBL data workflow. These medicinal chemistry journals frequently describe a drug discovery project and hence they typically contain data covering the assay types using in lead optimisation projects e.g. binding data on the primary biological target, data from cell-based assays, and ADMET assays for the same compounds. The investigation of scientific literature articles that consider *in vivo* assays within journals that have a toxicological or pharmacological focus may provide an additional source of relevant information, but this has not been explored as part of this work. If there is interest from the scientific community and it is considered to fall within the remit of ChEMBL, then this could be considered as a future task.

A new ‘assay classification’ table has been created within the ChEMBL database to store the annotated assay information. This table stores the hierarchical assay classification at three levels, and associated information:

- level 1 headings are broad categories of disease or phenotype;
- level 2 headings are groups of related diseases, phenotypes or toxicology annotation, and
- level 3 headings refer to a specific animal model or an endpoint with pharmacological or toxicological relevance.
- For each level 3 heading, associated information is given if relevant, and available, for:
- annotated MeSH terms,
- compounds that induce a specific animal model, and
- compounds that given a known outcome for a specific animal model (i.e. ‘standard’ or ‘positive control’ compounds).

The ‘assay classification’ table has a unique primary identifier (‘assay class id’) that maps (via an ‘assay id mapping’ table) to the ‘assay id’ given in the ‘assays’ table. In this way an assay (and its description) can be more mapped to more than one assay classification, if appropriate.

The *in vivo* assay dataset is available as a flat, downloadable file (Data Citation 1; see Usage Notes). The downloadable information includes:

- the dataset of annotated (and un-annotated) *in vivo* assays;
- the assay classification table of level 1, level 2 and level 3 headings with its associated information as described in the previous paragraphs.

## Technical Validation

Validation of the assay annotation has been carried out by comparison against 500 *in vivo* assays from ChEMBL examined by Zwierzyna and Overington^12^ where phrases have been manually assigned by database curators for experimentally induced animal disease models or phenotypes. For each matching *in vivo* assay, the reference animal model, or disease or phenotypic endpoint with pharmacological or toxicological relevance assigned in our work was compared against the annotation assigned by the database curators, as shown by the confusion matrix (Table 3) and classification statistics (Table 4). This shows that 315 *in vivo* assays are similarly annotated in our work (true positive), and 74 were similarly not annotated in our work (true negatives), with examples given in Table 5. The 63 false negative mismatches have a phrase in the assay description that has been identified by the database curators in^12^, but typically there is insufficient detail to accurately assign one reference animal model or a phenotype against the *in vivo* assay description (see the examples labelled ‘FN’ in the final column of Table 5). Equally, the 36 false positive mismatches typically have an annotated phenotype resulting from our work, but a similar phrase has not been assigned by the database curators (see the examples labelled ‘FP’ in the final column of Table 5). Overall, the validation comparison shows that the annotation of the descriptions of *in vivo* assays presents a reliable picture that can be used to match animal models described by the Hock publications or MeSH terms.

## Usage Notes

ChEMBL provides a number of mechanisms for searching and retrieval of relevant information (https://www.ebi.ac.uk/chembl/). The annotated dataset will initially be made available for download (Data Citation 1) but will also subsequently be accessible as part of a later release of the ChEMBL database, and via the web interface or web services (https://www.ebi.ac.uk/chembl/ws).

As explained in previous publications describing ChEMBL^2–4^, users should always be aware that although data are extracted manually and further curated, some errors are inevitable in such a large dataset and therefore data should always be treated with caution. For example, upon identifying an interesting endpoint within an *in vivo* assay, it is always prudent to consult the original publication to ascertain further details of the experimental procedures before using the data as the basis for further experiments.

## Additional information

**How to cite this article**: Hunter, F. M. I. *et al*. A large-scale dataset of *in vivo* pharmacology assay results. *Sci. Data*. 5:180230 doi: 10.1038/sdata.2018.230 (2018).

**Publisher’s note**: Springer Nature remains neutral with regard to jurisdictional claims in published maps and institutional affiliations.

## Supplementary Material



## Figures and Tables

**Table 1:** Examples of *in vivo* assay descriptions and annotation.

ChEMBL_ID	Assay description	Assay classification (Level 3)	Reference source or common term∗	Key∗∗
CHEMBL772714	Adjuvant arthritic rat activity determined with respect to spirogermanium at a dose of 30 mg/kg	Adjuvant Arthritis in Rats	Hock_2016	1
CHEMBL777832	Blood pressure lowering activity in renal hypertensive rats after intravenous administration; no drop in blood pressure or a statistically insignificant drop in blood pressure was observed (*in vivo*)	Renal Hypertension	Hock_2016	1
CHEMBL683996	Compound was evaluated *in vivo* for cysLT1 receptor induced airway obstruction in guinea pig (OA) at 2.0 hour after oral administration.	General Airway Models	phenotype	2
CHEMBL716118	*In vivo* antimalarial activity in mice (Mus musculus) against chloroquine-resistant Plasmodium yoelii species. NS after subcutaneous administration	General Antimalarial Activity	phenotype	2
CHEMBL732290	Analgesic activity in tail flick test, oral administration	Radiant Heat Method; General Analgesic Nociceptic and Allodynic Activity	Hock_2016; phenotype	3
CHEMBL723844	Compound was administered subcutaneously and was evaluated for opioid agonist activity by antinociceptive tail-flick (TF) assay in mice; I denotes Inactive at 30 mg/kg	Radiant Heat Method; General Analgesic Nociceptic and Allodynic Activity	Hock_2016; phenotype	3
CHEMBL2328414	Antioxidant activity against CCl4-induced oxidative hepatic injury Wistar albino rat model assessed as effect on liver cytosolic catalase activity per mg protein at 100 mg/kg, ip for 7 consecutive days prior to CCL4 challenge measured 24 h post CCl4 challenge (Rvb = 218.25 +/− 11.43 U/mg protein)	Carbon tetrachloride CCl4 Induced Liver Fibrosis in Rats; General Models of Drug Induced Liver Injury	Vogel_2008; phenotype	3
CHEMBL703119	*In vivo* antitumor activity against L1210 leukemia in 18 mice measured as T/C value; T/C = 18/9	General Leukemia; L1210 Experimental Leukemia; Neoplasms	Phenotype; Hock_2016; phenotype	3
CHEMBL785102	*In vivo* binding specificity the compound in rat striatum at 60 min of intravenous injection.	—	—	4
CHEMBL732935	Compound was evaluated for the time from injection to peak inhibition of the twitch response at 0.4 mg/kg dose	—	—	4

^∗^Hock_2016 is reference^18^, Vogel_2008 is reference^17^, ‘phenotype’ denotes that a common disease or phenotypic term can be identified within the assay description and annotated as such for e.g. ‘anti-Alzheimers’, ‘analgesia’, ‘inflammation’, ‘hepatotoxicity’).

^∗∗^Key to final column 1: A reference animal model can be identified from the assay description and annotated by a MeSH term. 2: A disease or phenotypic endpoint with pharmacological or toxicological relevance can be identified from the assay description and annotated by a MeSH term, but there is no direct match to a specific reference animal model. 3: Both a reference animal model AND a disease or phenotypic endpoint with pharmacological or toxicological relevance can be identified from the assay description. 4: No specific reference animal model or a disease or phenotypic endpoint with pharmacological or toxicological relevance can be identified from the assay description.

**Table 2:** Examples of mapping between MeSH terms and an individual reference animal model or a disease or an endpoint with pharmacological or toxicological relevance (‘phenotype’) in the ‘Reference Source’ column.

Assay classification Level 1	Assay classification Level 2	Assay classification Level 3	Reference source or common term∗	MeSH term(s)
Cardiovascular System	Cardiovascular analysis	Angiotensin II Antagonism *in Vivo*	Hock_2016	HYPERTENSION; CARDIOVASCULAR DISEASES; VASCULAR DISEASES
Nervous System	Anti-Epileptic Activity	General Anti-Epileptic Activity	phenotype	EPILEPSY; SEIZURES
Nervous System	Tests for Anxiolytic Activity	Acoustic Startle Response in Rats	Hock_2016	ANXIETY; REFLEX, STARTLE
Nervous System	Learning and Memory	Spatial Discrimination Learning in the Radial Arm Maze	Vogel_2008	MEMORY DISORDERS
Nervous System	Peripheral Analgesic Activity	Writhing Test for Analgesic Activity	Hock_2016	PAIN
Musculo-skeletal System	Anti-Inflammatory Activity	Paw Edema Test	Hock_2016	INFLAMMATION
Antineoplastic and Immunomodulating Agents	Methods for Testing Immunological Factors	Spontaneous Autoimmune Diseases In Animals	Vogel_2008	AUTOIMMUNE DISEASES; IMMUNE SYSTEM DISEASES
Alimentary Tract and Metabolism	Intestinal Function	Experimental Colitis Inflammatory Gut Disease	Vogel_2008	COLITIS, ULCERATIVE; INFLAMMATORY BOWEL DISEASES; CROHN DISEASE; DIGESTIVE SYSTEM DISEASES
Alimentary Tract and Metabolism	Liver Function	General Models of Drug Induced Liver Injury	phenotype	LIVER DISEASES
Alimentary Tract and Metabolism	Measurement of Blood Glucose-Lowering and Antidiabetic Activity	Anti-Diabetic Effects of Liver X Receptor Agonists	Vogel_2008	DIABETES MELLITUS; DIABETES MELLITUS, TYPE 2; DIABETES MELLITUS, TYPE 1
Genito Urinary System and Sex Hormones	Ovarian Hormones	General Estrogen or Progestogen Activity	phenotype	GONADAL HORMONES
Cardiovascular System	Cardiovascular Safety Pharmacology	Cardiovascular Safety Pharmacology: Mean Blood Pressure or Mean Arteral Blood Pressure	Vogel_2013	BLOOD PRESSURE
Antineoplastic and Immunomodulating Agents	Carcinoma Oncology Models	Lewis Lung Carcinoma	phenotype	CARCINOMA, LEWIS LUNG; CARCINOMA; NEOPLASMS, EXPERIMENTAL

^∗^Hock_2016 is reference^18^, Vogel_2008 is reference^17^, Vogel_2013 is reference^19^, ‘phenotype’ denotes that a common disease or phenotypic term can be identified within the assay description and annotated as such for e.g. ‘anti-Alzheimers’, ‘analgsia’, ‘inflammation’, ‘hepatotoxicity’) Note that some reference animal models or disease or phenotype are mapped to multiple MeSH terms.

**Table 3:** Confusion matrix for annotation of a set of *in vivo* assays.

n=488 assays		Predicted (this work)	
Positive	Negative		
Actual (from^12^)	Positive	TP=315	FN=63
	Negative	FP=36	TN=74
There are 488 identical *in vivo* assays with phrases that were manually assigned by database curators^12^ (‘actual’) in comparison to the disease/phenotype or reference animal model annotation performed in this work (‘predicted’). The remaining 12 assays (out of 500) had been classified as *in vivo* by Zwierzyna & Overington^12^ but are in fact considered to be *in vitro* or *ex vivo* assays.			

**Table 4:** Classification statistics for annotation of the 488 *in vivo* assays.

	Classification statistics
Sensitivity (TP/(TP + FN))	0.83
Specificity (TN/(TN + FP))	0.67
Precision (TP/(TP + FP))	0.90
F1 Score (2^∗^TP/(2^∗^TP + FP + FN))	0.86

**Table 5:** Examples of the set of *in vivo* assays for each quadrant of the confusion matrix.

**ChEMBL_ID**	**Assay description**	**Annotation carried out in this work**		**Phrase identified by database curators in** ^12^		**Statistics**
		**Animal model(s) or phenotype(s)**	**MeSH term(s)**	**Experiment**	**Phenotype**	
CHEMBL985321	Antiamnesic activity in scopolamine-induced mouse assessed as latency time to enter dark room in retention session at 10 mg/kg, ip treated 20 min before training session by passive avoidance test	Scopolamine Induced Amnesia in Mice Inhibitory-Avoidance Learning; General Inhibitory (Passive) Avoidance Learning; General Learning and Memory Models	MEMORY DISORDERS	avoidance test; scopolamine-induced; passive avoidance test	—	TP
CHEMBL1048342	Antidepressant-like activity in NMRI mouse assessed as reduction in immobility time at 0.01 mg/kg, ip by forced swimming test	Despair Swim Test; General Anti-Depressant Activity	DEPRESSIVE DISORDER; MENTAL DISORDERS	forced swimming test	Immobility; swimming	TP
CHEMBL1212532	Antiinflammatory activity in Albino rat assessed as reduction of carrageenan-induced paw volume at 20 mg/kg, po administered 1 h before carrageenan challenge measured after 24 h (Rvb = 1.39 +/− 0.053 ml)	Paw Edema Test; General Anti-Inflammatory Models	INFLAMMATION	carrageenan-induced; carrageenan challenge; [cPP1to7, NPY19 to 23, Ala31, Aib32, Gln42]-induced	paw volume	TP
CHEMBL2149100	Hepatoprotective activity against CCL4-induced liver damage in ICR mouse assessed as reduction in CCL-induced iNOS mRNA expression at 100 mg/kg, ip dosed 30 min before and 2 h post CCL4 challenge and measured 24 h post CCL4 challenge by RT-PCR	Carbon tetrachloride CCl4 Induced Liver Fibrosis in Rats; General Models of Hepatotoxic and Hepatoprotective Activity	LIVER CIRRHOSIS; LIVER DISEASES	CCL4-induced; CCL4 challenge; CCL-induced; [cPP1to7, NPY19 to 23, Ala31, Aib32, Gln42]-induced	liver damage	TP
CHEMBL733509	Median T/C calculated based on survivors at 300 mg/kg (318 umol) per day against Ip-implanted L1210 lymphoid leukemia mice	General Leukemia	LEUKEMIA	Ip-implanted L1210 lymphoid leukemia	Leukemia; lymphoid leukemia	TP
CHEMBL843578	Percent reduction was determined by using the ratio of mean of treated animal to that of control animal at a dose of 23.5 mg/kg	—	—	—	—	TN
CHEMBL825754	Number of rats with greater than 100% GH increase over the controlgroup, there are five rats in both control and compound treated groups.	—	—	—	—	TN
CHEMBL851918	Delta HR ratio measured as the ratio of delta HR(20 min)/delta HR (5 min)	Heart Rate Measurement	HEART RATE	—	—	FP
CHEMBL1820580	Hypolipidemic activity in Swiss albino mouse assessed as decrease in plasma triglyceride level at 50 mg/kg, po administered daily for 8 days measured on day 9 by spectrophotometry relative to control	Hypolipidemic Activity in Rats; General Lipid Metabolism	LIPID METABOLISM DISORDERS; HYPERLIPIDEMIAS	—	—	FP
CHEMBL726986	Analgesic activity of compound (5.31 + diprenorphinen M) in mice after icv administration	General Analgesic Nociceptic and Allodynic Activity	PAIN	—	—	FP
CHEMBL2020189	Inhibition of PI3K-mediated AKT Ser473 phosphorylation in human A2780 cells xenografted in nu/nu mouse at 10 mg/kg, po after 10 h by immunoblotting	—	—	human A2780 cells xenografted	—	FN
CHEMBL786334	Effect was expressed as mortality after injection of kainic acid (10 mg/kg) and the drug at the dose of 40 mg/Kg after 48 h	—	—	injection of kainic acid	—	FN
CHEMBL773481	Change in rectal temperature induced by DiPr-5,6-ADTN at a dose of 3.2 umol/kg of compound by subcutaneous administration	—	—	induced by DiPr-5,6-ADTN	rectal temperature	FN
Key to the final column TP: A similar phrase(s) has been identified by database curators in^12^ and annotated in this work. TN: No phrase has been identified by database curators in^12^ nor annotated in this work. FN: The assay description is not clear enough to accurately annotate a specific animal model or a disease or phenotypic endpoint, even though a phrase has been identified by database curators in^12^. FP: A phrase related to an animal model or a disease or phenotype has not been identified by database curators in^12^ but has been annotated in this work.						
